# Pediatrician’s perspectives on discharge against medical advice (DAMA) among pediatric patients: a qualitative study

**DOI:** 10.1186/1471-2431-12-75

**Published:** 2012-06-18

**Authors:** Bernadette C Macrohon

**Affiliations:** 1 Department of Pediatrics, Zamboanga City Medical Center, Zamboanga City, Philippines

## Abstract

**Background:**

The phenomenon of discharge against medical advice (DAMA) among pediatric patients places pediatricians in a dilemma between respect for the parent’s decision and the desire to provide complete care for the vulnerable child-patient. Little has been written about factors that affect a pediatrician’s decision to allow a parent to discharge his child against medical advice. This qualitative study aims to answer the question of how pediatric residents in a tertiary government hospital perceive and decide on a DAMA request from a parent or primary caregiver.

**Methods:**

Using a focus group discussion approach, 11 pediatric residents from a government-run tertiary hospital were recruited for the study. The session was digitally recorded and dominant themes were coded and identified.

**Results:**

There were three prominent themes that arose in the discussion: variability of definitions of DAMA, factors considered before “allowing” the patient to be DAMA, and the implications of a DAMA request on their performance as pediatricians. Definitions vary from one resident to another based on the main reason for DAMA (terminal, cultural, or financial). A conflict was noted in the definition of Home per Request (HPR) versus DAMA. Factors that influence a pediatrician to sign out a case as DAMA include: their ability to do something about the reason given for the DAMA request, the condition of the patient when the DAMA request was given, their impression of the kind of care that the parents provide, and their legal liabilities. Pediatric residents generally maintain a positive attitude towards the parents who request for DAMA and in the event of readmission, accept the patient into their care again.

The occurrence of a variety of definitions and subcategories for DAMA may cause confusion among the pediatricians and should be clarified. The familiarity with cultural traditions contributes to their ability to handle situations that may lead to DAMA but this should always be considered in the context of the pediatrician’s legal liabilities. The attitude of being helpful in spite of readmission after DAMA is an important attribute to be encouraged among new trainees.

**Conclusion:**

In most reviews about the phenomenon of DAMA, patient characteristics have been identified that make them prone to request for DAMA however; physicians also experience a complex process of decision-making in DAMA situations. It is therefore vital for every training institution to include ethical, legal and moral aspects of learning into their training programs especially in dealing with cases of DAMA.

## Background

The phenomenon of discharge against medical advice (DAMA) involves a situation wherein a patient chooses to leave the hospital before the attending physician recommends discharge [1]. This occurs not only in situations where the decision-maker is the patient himself but also in situations where the decision-maker is the main caregiver of the patient, such as in elderly or pediatric patients. Among adults, the rates of DAMA vary in different groups, from around 1–2% among psychiatric patients [1,2] to as high as 13% among HIV-positive patients [3]. DAMA conditions among the pediatric population poses a different challenge since children often are not considered to have the emotional and cognitive maturity as well as the legal rights to decide for themselves on such matters and therefore are dependent on their parents or legal guardians to decide whether to stay in the hospital or to be DAMA. A physician is bound by the Hippocratic oath to uphold the welfare of his patient at all times. However, in cases involving children, sometimes the pediatrician is caught between deciding on what he thinks is good for the child and the decision given by the parents which may be contrary to his appraisal. DAMA rates in the pediatric population vary between and even within a country. For example, in Iran, a pediatric center recorded a DAMA rate of 5.3% [4] while in Singapore’s Alexandra Hospital, a DAMA rate of 2% was seen [5]. In Nigeria, DAMA rates were recorded from 1.5% to 5.7% [6-8] among different pediatric centers. Also, a variance over time was observed: In the Zamboanga City Medical Center’s (ZCMC) Department of Pediatrics the DAMA rate was recorded at 2.1% in 2005 but rose to 4.6% for the first six months of 2010.

Common reasons for DAMA include a parent’s perception that the child is well, financial constraints, inconvenience of hospitalization (lost days at work, inability to care for other children), dissatisfaction of management (medical staff dissatisfaction), preference for traditional forms of treatment, and hopelessness of the clinical situation [4-8]. While many studies among adults have focused on patient characteristics that will predict DAMA such as the male sex [9,10] (although another study mentioned females [3]), younger age [3,9,10], non-White [9-11], being an IV drug user [3], being admitted in a non-teaching hospital [10], and receiving welfare on the day of DAMA [3], little has been mentioned about how physicians experience and perceive such a request to be DAMA. However, there are indications in the literature that the relationship between the physician and the patient plays a role for the likelihood of signing out as DAMA. For example, in Weingart’s report on the demographic and clinical profile of patients who were DAMA from a general medicine service in Boston’s Beth Israel Hospital, it was shown that there was significantly more patients without a personal attending physician who were DAMA versus those who had a personal attending physician (48.5% vs. 17.6%, *p* < 0.001) [9]. This factor was also mentioned in Alfandre’s review [1] on DAMA based on a study by Jeremiah et al. where the lack of a primary care physician in the outpatient setting was associated with a higher risk of signing out as DAMA. He further elaborated in this review that physicians should recognize overt signs of emotional distress that patients often exhibit prior to expressing their decision to DAMA. Physicians who are able to recognize these signs and address the patient’s underlying emotions can result in improved physician-patient relationship thus improving quality of care. This concept leads to the theory that the decision to DAMA may also depend to a large degree on the physician-patient relationship. More specifically, physicians’ perceptions and interpretations of a patient’s or a caregiver’s decision to be DAMA and the implications for their response to the situation are assumed to play a relevant role.

The consequences of DAMA on the pediatric patient is well documented, with observed readmission rates considered high from 20.7% to 24.5% [7,12,13]. Readmission produces complications such as higher costs of medical care [14] because of deterioration of the patient’s clinical condition at home. In a training institution catering to the low-income sector of society, the pediatric residents of the Zamboanga City Medical Center are often faced with a variety of reasons among the parents who decide to DAMA. However, their response to these factors will determine how they will tackle such cases of DAMA to prevent readmission and its complications.

This study therefore aims to answer the question of how pediatric residents in a tertiary government health care institution perceive and decide on a DAMA request from a parent or primary caregiver. Understanding the issues and concepts behind the process of deciding on DAMA for a patient whose fate depends largely on caregivers can help explain factors from the physician’s perspective that can be addressed to modify the outcome and improve the quality of care for this vulnerable group.

## Methods

### Research design

This is a qualitative study that aims to describe how pediatric residents of ZCMC perceive and decide on a DAMA request given by a primary caregiver on behalf of a vulnerable child. Since each patient in the ZCMC pediatric ward is attended by at least two pediatric residents, a focus group discussion approach was used to show the interaction among the pediatric residents during the decision-making process of signing out a case as DAMA.

### Research setting

The ZCMC Department of Pediatrics is a part of the national government agency Department of Health. The ZCMC also includes the Departments of Internal Medicine, Surgery, Obstetrics and Gynecology, Radiology, Anesthesia, Psychiatry, Pathology, Family Medicine, Rehabilitation Medicine, and Ophthalmology. The Department of Pediatrics building was designed to have an 80-bed capacity and is situated within the medical center compound. About 3000 to 3500 admissions to the department are recorded every year exceeding the expected bed capacity. The Zamboanga City Medical Center is the only institution in the entire southwestern Mindanao that has a pediatric residency training program. The Department of Pediatrics is headed by a Department Chair, a Training Officer, and a Chief Resident. An 11-person resident physician team undergoing pediatric training residency is supervised by 8 consultants with different sub-specialties in gastroenterology, nephrology, neurology, hematology and oncology, neonatology, pulmonology, cardiology, and ambulatory pediatrics. The admissions are directly handled by an assigned attending physician team. There are four teams of physicians. Each team is composed of two to three pediatric residents with 1 senior resident belonging to the late second-year to third-year level of training, and 1 to 2 junior residents of the first-year to early second-year level training. Each team is supervised by two consultants.

The Department of Pediatrics accepts newborn patients and those under the age of 14, after which they are transferred to the care of the Department of Internal Medicine. The ZCMC caters to families belonging to the lowest income groups and to various ethnic minorities in the region. The national dialect of the Philippines is Tagalog. The most common ethnic tribes in southwestern Mindanao include the Tausug, Visaya, Chavacano, and Samal. Other smaller tribes such as the Yakan, Subanen, and Badjaos and other ethnicities from other regions of the Philippines also receive treatment in the ZCMC. Some pediatricians belong to one or more of these tribes (because of intermarriages) and are able to speak several dialects.

### Subjects and sampling method

A purposive type of sampling was utilized with all 11 pediatric residents in training invited to join the focus group discussion for the study. No renumeration in cash or kind was given.

### Ethical considerations

The study protocol was submitted for review and approved by the Zamboanga City Medical Center Institutional Ethics Review Board (IERB) of which the author was the vice chairman. In order to avoid a conflict of interest, the author inhibited herself from the discussions of the rest of the IERB during the process of review. The pediatric residents were informed of the study protocol one month prior to the focus group discussion. They were likewise informed that their participation or non-participation, answers to the questions, and reactions regarding the issue discussed had no bearing on their assessments as pediatric residents. Moreover, while they were informed that the session was being recorded, it was assured that the audio recording and transcript will not reflect the identity of any specific resident who expressed his/her opinion to ensure confidentiality. This research study adheres to the RATS guidelines on qualitative research.

### Data collection

All pediatric residents were invited to join the focus group discussion over lunch provided by the investigator one Saturday afternoon, to avoid interference with the scheduled conferences during the work week. The author explained to the pediatric residents the objectives of the research and emphasized that they were allowed to step out of the discussion anytime. It was also emphasized at the start of the discussion that their answers would not in any way affect their performance evaluations. The following central questions were used to ask the residents about their experiences and perceptions regarding DAMA.

1. What does the status “Discharge against Medical Advice” mean to you?

2. How do you feel when a patient or parent requests to be Discharged Against Medical Advice?

3. What are some mechanisms that you use to convince them to stay?

4. When the patient comes back for re-admission, how do you feel about handling him again?

The focus group discussion started with the author asking the first question. The discussion was conducted in several dialects (Tagalog, Visaya, Tausug, Chavacano) mixed with English. There was a spontaneous and lively exchange of answers amongst the pediatric residents. When the respondents answered one question with the same answers or no new answers or concepts were introduced, the next question was introduced. The focus group discussion lasted for 76 minutes and was recorded with a digital recorder and was later transcribed verbatim by the author. One-on-one in-depth interviews were not performed since the participants were very open during the discussion and issues were clarified during the focus group discussion.

### Analysis

The transcripts were reviewed and themes identified through deduction and induction. Hypotheses about perception and factors that involve DAMA were synthesised from the review of literature. Based on the discussion, various issues or concepts about DAMA were noted and grouped under different previously identified hypotheses. In cases where a new issue or concept was raised, a new category or hypothesis was formed. These were then labeled under a common characteristic which is a theme. Dominant themes were then identified and refined. An issue or issues discussed under a recurrent theme was considered “dominant” and listed as a theme. Subcategories of themes or separation into two or more themes were done after re-assessment for independence of topic. The themes were reviewed by an independent research mentor. In cases of conflict, face to face discussions were done until resolution was achieved. The themes were then compared with established themes from other researches.

## Results

All 11 pediatric residents joined the focus group discussion. The level of training of the residents consisted of 2 third-year level trainees (most senior), 4 second-year level trainees, and 4 first-year level trainees. The Chief Resident also joined in the discussion. There were 8 female and three male pediatric residents. The ethnicity of the pediatric residents consisted of a mix of Tausug, Visayan, Tagalog, Chavacano, and Samal.

During the discussion, one of them had to step out during the early part because a patient at the ward needed medical attention. She promptly returned in a few minutes and joined in the discussion. There were three prominent themes that arose in the discussion: variability in the definitions of DAMA, factors considered before “allowing” the patient to go on DAMA, and the implications of a DAMA request on their performance as pediatricians.

### Definition of DAMA

In cases of DAMA, the pediatric residents have three main classifications or categories for DAMA which they also report in their monthly and annual census of admissions and discharges. Their decision as to the classification of the DAMA status depends on the reason given by the caregiver for the DAMA request, such as financial reasons (DAMA-financial), cultural reasons (DAMA-cultural), or terminal cases (DAMA-terminal). Cases signed out as DAMA-Financial are those cases where parents express the desire to be DAMA because of inability to shoulder expenses for medical treatment, inability to provide in-hospital care to their child because of lost days at work, or situations where the parents give other reasons such as bringing the child to a traditional healer since it is a cheaper form of treatment. However, this last reason overlaps with another common reason for DAMA that is to bring the child for traditional healing, the term of which they use is DAMA-Cultural, since this is based on cultural beliefs *(“…we know that the patient will die weeks or months after and they want to bring the patient to the traditional healer, so we label them as DAMA cultural”)*. The pediatricians believe that even though resorting to traditional healers is a common practice in this part of the country, going to traditional healers is also cheaper. To determine the cause for the DAMA request, much is based on the pediatrician’s own observation. Some of them observed that in cases with families who do not request for DAMA but wanted to consult a traditional healer, they would ask the healer to come over to the hospital to conduct a session of healing at the bedside. In situations like these, the pediatricians often allow the parents to bring traditional healers as respect to their cultural beliefs. The status of DAMA-Terminal, on the other hand, is given to a case where the child has a serious condition that has a poor prognosis of which the residents can’t do much to reverse the situation and out of hopelessness, the parents decide to bring the child home *(“As long as they [parents] know that the patient will die right after or immediately on discharge, [we classify as] terminal”)*. Amongst a subgroup of pediatric residents, a discharge status called Home Per Request or HPR is sometimes used. In this situation, the parent or caregiver still initiates the request to be discharged but the perceived prognosis of that particular case is good in contrast to DAMA where the perceived prognosis is poor *(“…the physician cannot trust the caregivers to administer the medications correctly or religiously so that’s when the physician terms it as DAMA. And if it’s like home per request, the physician trusts the parents and he knows that the parents know how to give the drugs so he can let go of the patient…more confidently, although the medications have not been completed and the patient is not completely well”)*. Differentiating one type of DAMA from the others would give the resident an opportunity to identify situations where they could still do something to help the family or to identify areas of care that need to be improved.

### Factors considered before giving the DAMA order

There are four major factors that a pediatric resident considers in deciding to write out a DAMA order: their ability to do something about the reason given for the DAMA request, the condition of the patient when the DAMA request was given, their impression of the kind of care that the parents/guardians provide, and their legal liabilities.

In general, the pediatricians’ ability to modify the situation that prompted the request for DAMA is one of the strongest factors that they consider when they sign out a case as DAMA. For example, when confronted with a request from a parent or guardian to DAMA, the participants in this study stated that the first thing they think about is the reason behind such a request. Their main motive for this is to determine whether they can still do something to help with the situation and allow the child to receive complete treatment *(“So we can find ways to stop them, for example, if the reason is financial, you can give options on how to solve the problem”)*. For this aim, they sometimes are required to interpret a possible “hidden” reason for the parents asking for DAMA because in their view the child is already well. This occurs when the child shows early favorable responses to the treatment regimen such as in very severe pneumonia cases. Children with this problem come into the hospital with fever, rapid and difficult breathing, cyanosis, and sometimes with decreased level of sensorium. After starting antibiotics, when the child’s fever lyses and is more awake, parents feel that their child has improved and opt to be DAMA rather than to complete the full course of antibiotics *(“It is their perception, Doc, that when they see that the patient is well clinically, the situation is okay. They rely on their own judgement that the patient is okay)*. However, the pediatricians believe that this is because of financial difficulties of having to buy the entire course of antibiotics, missed days of work (because laborers are paid according to a completed day of work), and additional costs of staying in the hospital like food and transportation expenses *(“…even though we tell the parents that we will shoulder the cost of the medicines and the hospital bill, they still want to go home because the parents claim that they don’t have money for food and other expenses like transportation during the hospital stay” or “the other parents can’t go to work because they had to stand watch in the hospital…”)*. Moreover, it was noted in the discussion that since the billing system changed in the hospital from 2005 up to 2010, it was harder for the patients to pay for their bills, especially among the poorest who used to get free medical care but are now required to shoulder a portion of the bill *(“…the patients are always surprised that medical services are not free anymore in this hospital”)*. There was no perception that there was a lack of comprehension on the part of the parents about the need for complete treatment, as the pediatricians reported to speak a variety of local dialects and are diligent with explaining the situation to the parents *(“....everybody explains to the parents, Doc, or maybe, we can have a longer explanation or have a family conference for better understanding.....” and “You cannot insist that they follow what you want so you’ll just need to explain very well”)*. There was unanimous agreement among the participants that if they perceive financial difficulty as the main reason for DAMA, they try to dissuade the family and instead try to look for ways to help them such as referring to the social services department, to the Philippine Charity Sweepstakes Office for grants, other private non-governmental organizations, or the pediatricians themselves shell out money for medications. If the reason for DAMA is modifiable by the pediatricians, their first recourse is to fix the situation in favor of the parents to alleviate the difficulties in continuing treatment. When the pediatricians feel like they can’t do much to help their patient’s family, then they sign out the case as DAMA.

In some instances, the pediatricians believe that one of the reasons for asking for DAMA is the “nurse factor”. This factor, which pediatricians believe can be modified, affects DAMA rates in two ways. Firstly, all the pediatricians believe that there are some nurses who encourage parents to bring home their children because this would mean less work for them. When the pediatricians were asked if they adapt the same attitude as the nurses, none agreed. Instead, one pediatrician explained that they view this situation of patient overload as a part of their job therefore, they shouldn’t be sending patients away. They also tried to share this attitude with the nurses who have been identified by the parents to have encouraged them to DAMA, to influence them not to encourage parents to DAMA *(“We tell the nurses not to think that way because we are basically being paid by the patient to do our work here”)*. The second way the “nurse factor” causes DAMA is dissatisfaction with nursing care. This is perceived by the pediatricians through such comments from parents that a few of the nurses are nasty, short-tempered, and procrastinate especially with IV catheter reinsertions *(“the parents don’t like the nurse-on-duty because she is nasty”, “the nurses scold the parents”, or “iv catheters are often dislodged and it takes a long time for the nurses to re-insert…..”)*. Again, if these are the reasons for DAMA, the pediatricians dissuade the parents from bringing their children out of the hospital and instead try to talk to the nurses to provide better service. None of the pediatricians mentioned that DAMA could be due to dissatisfaction with physician care, although one mentioned that she did wonder at one time if such a reason has been given about her services but was not fed back to her *(“I don’t know whether families under other pediatricians who were DAMA during my duty mentioned that they didn’t like me but among my patients who were DAMA, no one told me that they were dissatisfied.”)*. Another narrated that one of her patients requested to DAMA because the mother wanted to be under the service of a certain private pediatrician. She told the resident that their decision is not because of the pediatric resident’s fault, except that the child is “more accustomed” to the preferred private pediatrician *(“Because my child gets well all the time under Dr......., he is accustomed to that doctor”)*. Some interpreted this as a better way of telling the pediatric resident that they don’t like her management of the case but most believed that there was no reason for parents to be dissatisfied with their care.

The second major factor that influences the residents’ decision-making to sign out the case as DAMA is the medical condition of the child on the day of DAMA. When they see that the child is medically unstable such as those admitted in the intensive care unit or they feel that the chances of recovery is very slim if brought out of the hospital, the pediatricians sign out as DAMA. If the child is medically stable and is eating enough and is simply completing treatment in the hospital, they do not sign out as DAMA but sign out the case as HPR. What eventually came out from the discussion was an apparent gray area of what the order of HPR versus DAMA implies *(“the department should make an operational definition so that there is only one definition among the residents”)*. All the pediatricians mentioned that the status of either DAMA or HPR eventually is used for their own purposes, indicating how medically serious the patient is at that time, especially during presentations in the monthly mortality and morbidity conference *(“It gives a sense of personal satisfaction on the part of the physician in that he was able to send the patient home with a chance to survive....”)*. Nevertheless, there was one who expressed that in essence, HPR is still a form of DAMA since it’s the parents or guardians who requested to be discharged under a situation where the attending physician is not ready to discharge the patient yet. There was also conflict in terms of the appropriate situations to “use” the term HPR versus the term DAMA. For example, for a terminal case of cancer where the parent requests discharge, some would sign out as HPR apparently because the resident feels that there is not much that they can do to help but the patient is stable. However, if the diagnosis is something like pneumonia where much can still be done, they sign out as DAMA but some residents again base their decisions on the status and prognosis of the case.

The third major factor affecting the decision to sign out a case as DAMA is the pediatrician’s perception of the kind of care that the patient receives from the parents or the guardians. The discussion of this particular issue brought forth a lot of emotions. Most have indicated that when they see that the parents try their best in looking for resources to have the child taken cared of or that they show genuine compassion towards the patient, they try as much as possible to help them and try to defer a DAMA order. However, if they perceive that the parents “couldn’t care less”, even if they ask only once to be discharged, the pediatricians tend to give a DAMA order right away as they feel that their efforts are of no use since after treatment, the parents probably won’t take care of the patient again at home *(“there are cases of DAMA that you’d feel guilty about and wonder what happened to the child after being sent home and there were those cases that you couldn’t care less” and “There were cases that I experienced where I pitied the child so much that I thought he would’ve been better off dead than to suffer under the care of his parents”)*. This topic caused a division among the residents during the discussion because when one mentioned a situation as an example, wherein she felt that the family did not really care whether the child gets well or not, another pediatrician begged to disagree and thought that the parents just did not express themselves properly.

The fourth major factor that residents consider is the legal aspect of discharging the patient. When they think that the family might come back with an accusatory tone or file a complaint or malpractice suit because the child’s medical condition deteriorated after discharge, then it is best to have them sign out as DAMA. When they think that the family is more amiable and less prone to complain, they sign out as HPR *(“it’s more for the security of the physicians because they know that if they let the patient go and the patient will die, and they do not let the caregivers sign it, if something happens, the watchers can always go back on their word and say we were discharged and our patient died.”)*. In their setting, many relatives can complain and threaten to harm the resident of which two cases were cited in the discussion. In one case, some relatives came back and requested for “blood money” (money paid by the physician or hospital to the family in exchange for the death of the child largely blamed on the physician) and threatened to physically harm the resident or a member of her family if not given. Another pediatrician described how the family approached the district congresswoman to ask for money to help with funeral costs after the death of their child. They claimed that they were sent home thinking that the child was well but eventually died within 24 hours, implying some form of mismanagement. When the congresswoman’s staff investigated the case, the attending pediatrician presented to them the signed DAMA request of the parents, thus absolving them from the case. The residents then concluded that to be able to protect themselves from matters like these, especially when they move on to private practice, it is best to sign out the case as DAMA and even for cases such as HPR, it might be best that they consider it as DAMA as well.

### Implications of a DAMA request on their performance as a pediatrician

From all the discussion, pediatric residents see that DAMA has a negative connotation wherein there is dissatisfaction of treatment outcome from the point of view of the parents (i.e. the effort that was put in does not equate to the improvement of the patient). From the point of view of the pediatricians, it is an expression of a sense of hopelessness in the management of the patient. All of the pediatric residents did not take the DAMA request personally and maintain an open and amiable attitude when faced with readmission of their patient. Given a case of readmission of a DAMA patient, the pediatricians unamimously said that they don’t mind treating the patient again *(“I will accept the patient again because the child is at the mercy of the caregivers and they cannot decide for themselves. If, for example, you had a misunderstanding with the grandparents or uncles or aunts, which was why they decided to DAMA and then they come back, I still have to put the patient’s best interest in mind.”)*. They also mentioned that they don’t feel that the previous DAMA status would affect their management of the case, although many have given side remarks of “I told you so….”. This attitude was explained by the pediatricians that in a government hospital, no patient is refused admission so they don’t have a choice who to take care of. Moreover, there is much emphasis on respecting tribal beliefs as well *([pertaining to the tribal group Badjao] “It’s like, [the Badjaos] let fate dictate what happens.... whatever will be, will be, if it’s time to go....they easily accept their fate”)* so they still accept the patient after being brought to a traditional healer and then returned back to the hospital. Nevertheless, most of them try to understand the situation that the family was put in and then try to approach them differently from before.

## Discussion

Discharging against medical advice (DAMA) is a known phenomenon in different settings around the world and is clearly defined among adult patients [1]. However, its application among the pediatric population is less clear. In the Zamboanga City Medical Center, the term DAMA apparently has different meanings and usage. Based on the focus group discussion, the pediatricians define DAMA as a request to bring a child home when the attending pediatrician hasn’t given the discharge order. However, in contrast to most definitions used elsewhere, the pediatricians required that the prognosis of the medical condition is poor. Based from the FGD, this requirement is used for census purposes (i.e. so that the residents know that this patient will most likely expire once brought home in contrast to someone who will most likely survive, labeled as HPR) rather than for legal purposes. In fact, only one pediatrician in the group was more aware of the same legal implication whether they send the patient home with good or poor prognosis. He stated that either way, the child was still brought home against the pediatrician’s better judgement and should be clearly stated in the record to be able to protect themselves.

This study identified three categories of DAMA that essentially covers most of the other established reasons for DAMA in other parts of the world [4-8], such as financial constraints, cultural beliefs, and terminal cases. There were no other categories of DAMA that indicate other possible reasons for the request, such as dissatisfaction of medical care, in spite of the pediatrician’s perception that poor nursing care may have initiated the request. The use of the term HPR or home per request by the pediatricians as a separate entity from DAMA is unique to this institution. The basis of its use is the attending pediatrician’s assessment that the outcome of care is good even if the parent requested to be discharged. This is a risky decision for the pediatrician because complete cure can never be guaranteed. This conflict of terms and their implications should be addressed to provide a uniform and clear definition for its use amongst the pediatricians and the hospital personnel.

It is interesting to note that most of the pediatricians did not consider dissatisfaction with physician care as a factor that might have prompted a request to DAMA. This should be an important insight as in some studies, one of the frequent causes of DAMA is dissatisfaction with physician care [4]. Failure to recognize this may give a false positive feedback to the pediatricians and thus prevent improvement in the way they manage their patients and their families. Another reason why this factor is not acknowledged as much among the pediatricians is because in the Filipino culture, development of close personal ties usually occurs between the patient’s family and the physicians, as evident in frequent invitations of the physicians to patients’ christening or birthday parties. Therefore, a strong physician-patient-family relationship is believed to be helpful in dealing with DAMA. Another practical reason for the perception that dissatisfaction with physician care is not a major factor for DAMA could be that parents do not want to antagonize the pediatricians or nurses because of the fear that the health care being provided to their child might be compromised in the future. And lastly, the participants belong to the same department in the same institution and therefore have developed close ties. In this situation, their perception of a complaint against a colleague may have been distorted to mean otherwise or may have been addressed directly by another person thus minimizing the impact of such a complaint.

Two factors that the pediatricians consider in giving the DAMA order are their perception of the kind of care that the child will receive at home and the attitude of the parents or caregiver towards the medical team. However, being a subjective parameter, a family may be misjudged by one pediatrician and may not be guided properly in the treatment of their child. This appraisal is underlined by the finding that two pediatricians described one situation differently during the focus group discussion. Therefore, in cases like this, it may be advantageous to have more than one pediatrician handling the case as this can provide a more objective view of the family’s reactions and perspectives.

When a patient is re-admitted after being discharged against medical advice, the pediatricians showed openness in accepting the patient. This is an important attitude to develop as it removes negative feelings and promotes trust between the family and the physician. Moreover, they maintain a helpful attitude in trying to mediate the situation to encourage parents to continue therapy until completion of treatment of their children.

There was only one pediatrician who emphasized the importance of signing the DAMA request as protection against malpractice complaints. While there has been no malpractice complaint filed against any pediatric resident during their training, there have been various types of complaints mentioned in the discussion. Therefore, the legal implications in signing out a case as DAMA should be taught among pediatric trainees. In addition, pediatric trainees should also be taught the legal responsibilities of a pediatrician in the light of being a protector of a child’s welfare.

This study discussed an important social aspect regarding pediatric care that is the phenomenon of DAMA. Being limited to resident pediatric trainees in a hospital catering to the low income group, the perspectives shared by the subjects in this study do not reflect those of private pediatric practitioners in Zamboanga City. Moreover, the income group in private hospitals is different from that of the group in this study, which may affect the reasons behind DAMA requests. Another limitation was noted in the analysis of the transcript. The identification of themes was limited to only two persons, the author and another person. This ensured up to a small degree the objectivity of the identified themes however, since only the author was able to review the transcripts fully, there may be some themes that may have been missed.

Further research may be done to compare the DAMA perspectives of all pediatricians in the country. Likewise, with the many different tribal groups in the entire country, various DAMA cultural factors may also be compared.

## Conclusion

In most reviews about the phenomenon of DAMA, patient characteristics have been identified that make them prone to request for DAMA however; pediatricians also experience a complex process of decision-making to sign out their cases as such. The decision of signing out a case as DAMA involves four main issues: the reasons behind the request for DAMA, their perception of the patient’s condition, their impression of the kind of care that the parents/guardians provide, and their legal liabilities. Physicians, specifically pediatricians, being in the service of taking care of the welfare of a vulnerable group (children), are often torn between watching out and fighting for what they think will be appropriate for their patients according to medical practice, giving in to the wishes of the parents and caregivers, and at the same time having to pay attention to their own protection against liabilities. It is therefore vital for every training institution to include ethical, legal and moral aspects of learning into their training programs especially in dealing with cases of DAMA.

## Abbreviations

DAMA, Discharge Against Medical Advice; ZCMC, Zamboanga City Medical Center; HPR, Home Per Request; IERB, Institutional Ethics Review Board.

## Competing interests

The author is a part-time consultant in the Department of Pediatrics of the Zamboanga City Medical Center. She assists in training the pediatric residents and is a neurology consultant for cases referred to the pediatric neurology service.

## Authors’ contributions

BM conceptualized, designed, collected data and wrote the entire manuscript.

## Funding

No funding was received for this study.

## Pre-publication history

The pre-publication history for this paper can be accessed here:

http://www.biomedcentral.com/1471-2431/12/75/prepub
